# Exploring the economics of public health intervention scale-up: a case study of the Supporting Healthy Image, Nutrition and Exercise (SHINE) cluster randomised controlled trial

**DOI:** 10.1186/s12889-022-13754-0

**Published:** 2022-07-14

**Authors:** Vicki Brown, Huong Tran, Joanne Williams, Rachel Laws, Marj Moodie

**Affiliations:** 1 Deakin University, Geelong, Deakin Health Economics, Institute for Health Transformation, Global Obesity Centre (GLOBE), School of Health and Social Development, Victoria, 3220 Australia; 2 School of Health Sciences, Swinburne University of Technology, Hawthorn, VIC 3122 Australia; 3 Deakin University, Geelong, Institute for Physical Activity and Nutrition, Victoria, 3220 Australia

**Keywords:** Economics, Implementation, Scale-up

## Abstract

**Background:**

The costs and benefits of an intervention within the intervention testing phase may differ from those experienced when that intervention is implemented and delivered at scale. Yet limited empirical work has been undertaken to explore how economic constructs related to implementation and scale-up might have an impact on intervention cost. The aim of this study was to explore the potential economic impacts of implementation and scale-up on a healthy weight and body image intervention tested in a Type II translational research trial.

**Methods:**

The Supporting Healthy Image, Nutrition and Exercise (SHINE) study is a cluster randomised controlled trial, aiming to deliver universal education about healthy nutrition, physical activity and wellbeing behaviours to adolescents in Australian secondary schools. Data on the cost of the intervention were collected alongside the trial using standard micro-costing techniques. Semi-structured interviews were conducted with key intervention stakeholders to explore the potential economic impacts of implementation and scale-up. Thematic content analysis was undertaken by two authors.

**Results:**

Fifteen intervention group schools participated in the 8-week online intervention targeting students in 2019 (99 Grade 7 classes; 2,240 students). Booster sessions were delivered during one class session in Grades 8 and 9, in 2020 and 2021 respectively. Time costs of intervention delivery and co-ordination comprised the majority (90%) of intervention cost as per the trial, along with costs associated with travel for intervention training and equipment. Themes related to the benefit of the intervention emerged from interviews with six intervention stakeholders, including the potential for economies of scale afforded by online delivery. Contextual themes that may have an impact on intervention implementation and scale included acceptability across all school sectors, availability and reliability of IT infrastructure for intervention delivery and variations in population characteristics. A number of key alterations to the intervention program emerged as important in supporting and sustaining intervention scale-up. In addition, significant implementation costs were identified if the intervention was to be successfully implemented at scale.

**Conclusions:**

The findings from this study provide important information relevant to decisions on progression to a Type III implementation trial, including budget allocation, and will inform modelled economic evaluation.

**Supplementary Information:**

The online version contains supplementary material available at 10.1186/s12889-022-13754-0.

## Introduction

Overweight and obesity is a pressing public health issue worldwide [1], requiring effective and cost-effective obesity prevention interventions delivered at scale to reduce the associated health and economic burden. Many obesity prevention interventions have been trialled internationally, with some demonstrating favourable results [2, 3]. Relatively few evidence-based obesity prevention interventions have however been successfully and sustainably implemented and delivered at scale to populations [4, 5]. This is despite the range of practical tools [4, 6], theories, models and frameworks [7, 8] available to guide, understand and evaluate intervention implementation and scale-up.

Economic-related facilitators and barriers to successful and sustainable intervention implementation and scale-up, such as workforce capacity and funding sustainability [7, 9], are commonly reported. It is well-recognised that both the costs and effects of an intervention experienced in the evidence-building stage (i.e. within efficacy trials) may differ to the costs and effects experienced when that intervention is implemented and delivered at scale in the “real world” [4, 5, 10]. Implementation and scale-up are complex, given changes in scope, delivery, dynamic effects through time and the impact of system effects. The costs and effects of a scaled-up intervention may vary from those experienced within a more controlled research setting, due to a range of contextual and other factors such as intervention design, the preferences of stakeholders and economic influences such as resource availability, workforce capability and funding [11]. For instance, in a research setting, research staff may be involved in delivering an intervention, while, in the ‘real world’, the workforce to deliver an intervention may differ, and be influenced by contextual factors such as the system in which the intervention is being implemented and the availability of resources.

Recently published evidence estimated a ‘scale-up penalty’ for obesity prevention interventions across measures of weight status, physical activity/sedentary behaviour and nutrition; the scaled-up intervention effect was typically less than 75% of that reported in the eight included efficacy trials [5]. Very limited evidence currently exists on the implications of scale on intervention costs, and when combined with information on effectiveness at scale, cost-effectiveness. The recent study by Roberts et al. [12] found that economic analysis currently appears to have a less important role in informing implementation decisions and in the appraisal of spread and scale within wider populations. Study findings highlighted the emergent application of economic analysis within implementation research, and that this area of research is still developing [12]. While best practice guidelines for conducting and reporting economic evaluations exist [13, 14], they do not currently provide detailed guidance on how to consider the economics of interventions as they are implemented and scaled up, moving from research to policy and practice.

To make informed decisions, policy-makers require evidence of the impact of implementation and scale-up on intervention costs, as both affordability and cost-effectiveness have been demonstrated to be key factors in successful and sustainable intervention scale-up [4, 7, 15]. Qualitative data collection methods, such as interviews, have previously been used to collect data to inform health economic research and have been recognised as rich sources of information [16]. For example, the WHO-STOPs systems-based obesity prevention intervention utilised key informant interviews to inform intervention costing alongside the research trial [17]. Recently, both quantitative and qualitative approaches to economic evaluation have been proposed as a means to better incorporate the impacts of implementation and scale into economic evidence [18]. While qualitative techniques have informed cost estimates alongside controlled research trials, they have been used less frequently to assess the potential cost impacts of intervention scalability and implementation as an intervention moves from research to practice.

The Supporting Healthy Image, Nutrition and Exercise (SHINE) study is a Type II translational cluster randomised controlled trial (cRCT), conducted in Melbourne, Australia from 2019 to 2022. Type II trials test the efficacy and effectiveness of an intervention, whereas Type III trials focus on implementation and dissemination research [19]. SHINE aims to deliver universal education about healthy nutrition, physical activity and wellbeing behaviours to adolescents (aged approximately twelve to thirteen years in 2019) in secondary schools, with randomisation at the school level. The intervention replaces the usual Health and Physical Education (HPE) classroom-based curriculum [20] for eight weeks and aims to improve body image, mental health, nutrition and physical activity and to help prevent the development or progression of overweight and obesity. The SHINE study incorporates a “within-trial” and modelled economic evaluation [21]. This means that the costs and consequences of the SHINE intervention have been collected exactly as they occurred within the controlled research environment. This ‘within trial’ data will then inform a modelled evaluation that extrapolates the decision problem to a wider population and across a longer time horizon [21].

This paper aims to: (i) quantitively estimate the within-trial intervention costs of the SHINE intervention from the public payer perspective; and (ii) to qualitatively explore the economic-related factors that may potentially influence the scale-up of the SHINE intervention. The predictive nature of this study will provide trialists and decision-makers with valuable economic information on the potential for scale- up of the SHINE intervention, and potential impacts on overall intervention affordability. This will also provide health economists and trialists with a case study example of how qualitative research methods might complement more traditional quantitative data collection and analysis in health economic evaluation, to generate economic evidence potentially relevant to implementation and scale. Finally, the evidence generated will help to inform the extrapolation of potential intervention costs for the modelled economic evaluation of the SHINE intervention [21].

## Methods

The SHINE cRCT is registered with the Australian New Zealand Clinical Trials Registry (#12618000330246).

### The SHINE intervention and randomised controlled trial

SHINE intervention content is based on an effective program originally developed for college students in the USA (the “Staying Fit” program [22, 23]), and modified as both a universal and targeted intervention for adolescents in the Australian context. The program consists of four themes on healthy habits—nutrition, physical activity, emotions and body (self) image, and each theme has multiple modules that can each be completed in approximately five minutes. Participants screened as experiencing or at-risk of having depression, eating disorders or weight issues receive targeted intervention content, surreptitiously delivered through individual pathways for completion so as to avoid potential stigma. Booster SHINE sessions are delivered to students during one class period in both Grades 8 and 9, one and two years after commencement of the program. Participating schools are offered in-person teacher training sessions on intervention delivery, on-going in-person or telephone support as required, and intervention resources such as training manuals and earbuds for students to engage with the online intervention content.

Schools were randomised to intervention or waitlist-control arms using concealed web-based randomisation. Ethics approval for passive consent was granted (Deakin University Human Research Ethics Committee (#2017–269); Victorian Department of Education and Training (#2018_003630)), enabling all students in Grade 7 at participating schools to receive either the intervention or the standard teacher-delivered curriculum. Students with written opt-out/non-consent from parents did not participate in the study. While the RCT is still underway and efficacy has not yet been estimated, data on the primary outcome of measured body mass index (BMI) and self-reported body dissatisfaction have been collected at baseline, post-intervention, 12 months, 24 months and will be collected at 36 months.

### Estimation of “within-trial” intervention cost

The cost of the SHINE intervention was collected alongside the cRCT from the public payer perspective [21]. The public payer perspective was chosen as both education and healthcare are publicly, universally provided in Australia. The intervention was costed as ‘steady state’, assuming that the intervention is fully operational and omitting costs associated with intervention development. Data on the cost of the intervention were collected between 2019 and 2021. Incremental costs from resource use associated with the intervention were identified using pathway analysis and standard micro-costing techniques [24]. Participating schools were offered in-person teacher training sessions on intervention delivery, and data related to travel and time costs associated with these sessions were collected from trial records and costed using published rates [25, 26]. Schools were also able to access in-person or telephone intervention delivery support throughout the course of the trial, and data related to these travel and time costs were also collected using a standardised tool created in Microsoft Excel. Annual booster sessions in Grades 8 and 9 were completed during one class, and the time cost for electronic reminders to schools to complete these booster sessions were also included. Costs of the provision of intervention-related equipment were identified from trial records and published values. Intervention administration costs were included, using published salary costs [25] and estimates of time for a Project Manager to assist in intervention delivery (as opposed to research-related activities). Project management time costs for the co-ordination of intervention delivery and provision of intervention support was included at 0.3 full-time equivalent (FTE) load for a Project Manager in year 1. In years 2 to 4, project management time costs were equivalent to 0.1FTE, to co-ordinate and support booster session delivery (i.e. send reminder emails to schools). A survey was also conducted with intervention group teachers, to ascertain time use in relation to lesson planning as compared to usual practice. The survey, conducted after the SHINE 8 week program in 2019, asked teachers to estimate the time spent planning SHINE HPE lessons and differences in time between planning SHINE HPE lessons and standard HPE curriculum lessons. Teachers could also provide free-form text on any additional feedback they had on the SHINE intervention more broadly. All costs were measured in 2021 Australian dollars (AUD) and discounted at the commonly accepted 3% discount rate [27].

#### Sensitivity analyses of intervention cost

Sensitivity analyses of intervention costs were examined, to estimate the effect of variation of key costing parameters on overall costing results. Sensitivity analysis included increased uptake in teacher in-person training to 100% of all participating teachers and exclusion of teacher time costs (i.e., assuming no difference in teacher time cost for lesson planning between SHINE HPE curriculum lessons and standard lessons).

### Qualitative interviews to inform estimation of scaled-up intervention cost

The qualitative component adopted a constructivist approach. Constructivist qualitative research aims to understand a phenomenon from the perspective of those experiencing it, with the researcher’s understanding co-constructed with that of the participants through their mutual interaction within the research setting and through data generating techniques [28].

Semi-structured interviews were conducted with key intervention stakeholders by one female author holding a PhD in health economics (VB). The interviewer had experience in conducting qualitative interviews, and undergraduate training in qualitative research methods. Key intervention stakeholders, including policy personnel (e.g. curriculum authority representatives), program deliverers (e.g. teachers) and lead intervention researchers (e.g. Chief/Associate Investigators, Project Manager) were purposively sampled and invited by email to participate in interviews conducted in their workplaces or online (via Zoom). Potential interview participants were identified through discussion with the lead researcher of the SHINE cRCT, so as to not impede the cRCT work. The proposed sample size of five policy personnel, five lead intervention researchers and ten program deliverers (e.g. the teachers directly involved in the SHINE cRCT intervention group) was also designed not to impede the cRCT work or introduce undue participant burden. While some of the lead intervention researchers may have had an established academic relationship with the interviewer, the policy personnel and program deliverers had no pre-established relationship with or knowledge of the interviewer.

Semi-structured interview guides were developed by one author (VB) and reviewed by an author with experience in implementation science (RL) to ensure comprehensibility (Additional File 1). These were guided by the Knowledge-To-Action framework [29], the Consolidated Framework for Implementation Research (CFIR) [30] and a review of economic constructs in commonly utilised implementation and scale-up theories, models and frameworks [31]. Interviews were recorded and transcribed by a professional transcription company verbatim, and all participants were deidentified and assigned a participant code.

Qualitative data analysis was a continuous process during data collection. Transcripts were uploaded to software QSR-NVivo 12 [32] to manage the data. Two authors (VB, HT) read all interview transcripts in their entirety to familiarise themselves with the data, following Braun and Clarke’s six step process for thematic analysis [33]. Thematic content analysis was undertaken, with the purpose of providing valid inferences from the data, new insights and a practical guide to action [34]. The development of initial codes was undertaken inductively and independently by two authors (VB, HT), and codes were compared between the authors. Codes were then abstracted into key themes deductively, using a codebook based on a framework of the economic constructs in commonly utilised implementation and scale-up theories, models and frameworks [31]. Each step of the coding process was discussed between the two authors (VB, HT). Qualitative findings were reported following the CORE-Q checklist for qualitative data [35] (Additional File 2). Common themes are presented in a table (Table 4), along with supporting quotes from participants and a brief summary of whether the theme may potentially have implications for intervention cost, benefit or uptake of the intervention (either as a barrier or enabler to intervention uptake) should it be delivered at scale.

## Results

### Within-trial cost of intervention

The SHINE RCT recruited 15 intervention group schools, with a total of 99 Grade 7 classes and 2,240 Grade 7 students participating in the 8-week online intervention in 2019 (Table 1). Booster sessions were delivered during one class session in Grades 8 and 9, in 2020 and 2021 respectively. In-person training and assistance in the intervention was delivered upon the request of three intervention schools (20%), involving an onsite one-hour meeting at the school with teachers involved in delivering the SHINE intervention (*n* = 18 in total), the SHINE Principal Investigator and/or a Research Assistant (Table 1). While the intervention content is delivered online, this training and assistance time was spent familiarising teachers with intervention content and the online delivery platform. Teacher training manuals were provided to a total of 84 teachers involved in intervention delivery and custom earbuds were given to all intervention students to facilitate engagement in the audio components of the online intervention (Table 1).

**Table 1:** Characteristics of intervention group schools

School characteristic	
**School system**	
Independent or Catholic school	6 (40%)
Government school	9 (60%)
**School structure**	
Co-educational	13 (87%)
Single-sex	2 (13%)
**Location**	
Metropolitan Melbourne	10 (67%)
Regional Victoria	5 (33%)
**School size**	
Mean number of Grade 7 classes per intervention school (range)	7 (2 – 10)
Socio-economic Index for Areas (SEIFA) Index of Relative Socio-Economic Disadvantage (IRSD)^*^	
*SEIFA Index*	*No. intervention schools*
2	1 (7%)
4	1 (7%)
5	1 (7%)
6	1 (7%)
7	4 (27%)
8	4 (27%)
9	2 (13%)
10	1 (7%)

^*^SEIFA IRSD summarises a range of information about the socio-economics of people and households within a local government area. SEIFA IRSD are presented for the LGAs in which intervention schools are located [36]. A lower score indicates relatively greater disadvantage

Twenty-nine intervention teachers completed the teacher time use survey (35% response rate from a total of 84 intervention group teachers). The majority of respondents reported taking less time to plan SHINE HPE lessons, as compared to usual HPE lessons (*n* = 20 teachers, 69%). Three teachers (10%) reported taking more time to plan SHINE HPE lessons as compared to usual HPE lessons, and six teachers (21%) reported no difference in planning time between SHINE HPE lessons and usual HPE lessons. When averaging all valid teacher responses, the mean time taken to plan SHINE HPE lessons compared to usual HPE curriculum lessons was a time-saving of 141 min over the eight weeks of intervention delivery (range 480 min saved to 60 min extra time taken; 24 of 29 valid participant responses, as five participants did not specify the estimated number of minutes lesson preparation time differed by). The total intervention cost was estimated to be $41,562, equating to an intervention cost of $19 per intervention group student (Table 2).

**Table 2:** Resource use and intervention costs collected alongside the SHINE cRCT

**Time cost**	
*Year 1*	
Project Management, intervention delivery co-ordination	$30 335
Teacher training and assistance	$1 910
Teacher time cost-savings	-$10 331
*Year 2*	
Project Management, intervention delivery co-ordination	$8 049
*Year 3*	
Project Management, intervention delivery co-ordination	$8 049
TOTAL TIME COST	$38 011
TOTAL TIME COST (discounted)	$37 315
**Travel costs**	
*Year 1*	
Travel to provide teacher training and assistance	$235
TOTAL TRAVEL COST	$235
**Equipment costs**	
*Year 1*	
Teacher training manuals	$428
Custom earbuds	$3 585
TOTAL EQUIPMENT COST	$4 013
TOTAL COST	$42 259
**TOTAL COST (discounted)**	**$41 562**
**TOTAL INTERVENTION COST PER STUDENT (discounted)**	**$19**

Costs are in 2021 Australian dollars. Negative values indicate cost-savings

In sensitivity analysis we assumed that teacher training and assistance was provided in a one hour in-person session with an intervention researcher to all schools, with all intervention group teachers participating. We also excluded teacher time costs for lesson planning, resulting in the total intervention cost rising to $57,550 ($26 per intervention student) (Additional File 3).

### Results from qualitative interviews

Study recruitment and in-person interviews began in February 2020. At this time, five lead researchers, three policy personnel and four program deliverers (i.e. teacher participants) had been successfully recruited to the study, and interviews had been scheduled to be conducted between February and June 2020 for these participants. In addition, a further 78 teacher participants and one policy personnel participant had been invited to participate (with 31 having already declined to participate, and 48 to be followed up at that time).The global pandemic was declared in March 2020, and Australia was forced into a six-week national lockdown followed by a series of state or citywide lockdowns in Melbourne, Victoria. Due to the pandemic, the Victorian Department of Education issued a directive that research involving schools be halted for several months in 2020 and again in 2021. Given this directive and the move to online learning (and associated teacher workloads), interviews with teacher participants in the SHINE intervention were not able to take place. In addition, two policy personnel participants withdrew from the study, citing significantly increased workloads due to the pandemic and lockdowns. Any further recruitment of policy personnel was halted at this time, so as to minimise burden on key contacts given the disruption at the time. Interviews were however conducted with intervention lead researchers (*n* = 5) and policy personnel (*n* = 1) between February and August 2020. Four of these interviews were conducted in-person, just before coronavirus restrictions, and two interviews were conducted during lockdown using online technology (i.e. Zoom). Average interview length was 32 min (range 12 to 49 min). In addition, 22 out of the 29 intervention teachers that completed the time use survey in 2019 also provided freeform text responses on feedback on the SHINE intervention. These responses were included into our coding and data analysis, using the pre-defined methodology for thematic analysis of the qualitative interviews, so as to provide some program deliverer perspectives from the limited data we were able to collect from this stakeholder group (Table 3).

**Table 3:** Description of participants in qualitative analysis of SHINE program

	**Total**
Total number of interviews	6
Intervention lead researchers	5 (3 female, 2 male)
Policy personnel	1 (1 female)
Free text survey responses from teachers	22 (gender not known)

#### Themes arising from qualitative data

Several themes were identified that may potentially impact on the cost (and cost-effectiveness) of the SHINE intervention if scaled-up. A qualitative results table is presented in Table 4, outlining key themes, supportive quotes and potential implications for cost-effectiveness of the intervention should it be delivered at scale.

**Table 4:** Qualitative results table detailing themes, supportive quotes and potential implications on cost or uptake of the intervention

Theme and sub-theme	Example supporting quote	Potent implications for
**Theme: Benefit of the intervention**		
Universal delivery	Researcher 03: “If schools were delivering the HPE curriculum to the level that it needs to be, there probably wouldn’t be much difference because the message is that foundation message of: what is healthy behaviour? But I feel that where SHINE has the advantage, it’s delivering the same message to everyone rather than getting perhaps a diluted message from various teachers of varying knowledge bases.”	Uptake of the intervention – potential enabler
Targeted intervention with universal delivery	Researcher 05: “I think that the students are able to get targeted messages in a way that doesn't appear targeted at all is probably the best aspect of it. So that way, they can work through the program at their own pace and pick what's interesting for them, but then also get some additional information that may help with some underlying problems.”	Uptake of the intervention – potential enabler
**Theme: Economies of scale**		
Economies of scale	Researcher 05: “I think that the students are able to get targeted messages in a way that doesn't appear targeted at all is probably the best aspect of it. So that way, they can work through the program at their own pace and pick what's interesting for them, but then also get some additional information that may help with some underlying problems.”	Cost – potential for cost-savings through economies of scale
**Theme: Acceptability, accessibility**		
Aligns with curriculum	Researcher 04: “…we contracted a health and physical education curriculum expert to review the program content to make sure that it did align with the junior years … Basically, the curriculum’s done for the teachers, all they have to do is just monitor.”	Uptake of the intervention – potential enabler
Busy nature of the school environment, need to integrate into existing work practices	Policy personnel 06: “I think, in terms—if schools don’t see it as an added extra, you’ve got greater leverage. Schools are asked to do a lot of things. Schools are asked to do respectful relationship program. They’re asked to do—they’ve got to track kids against their activity levels, their resilience levels, are they happy, healthy and well. The government ask them to do a lot of things. If you can show that it’s not an add on, I think you’ve got a feasible program. If it can be done with limited resourcing within the school, then you’ve probably got a feasible program.”	Uptake of the intervention – potential barrier
Sensitive content	Researcher 01: “…you can’t go in to Catholic schools because they felt that Year 7 was too young to be addressing the eating disorder issue. Despite the fact being that the most common age for the onset of eating disorder is twelve, which is exactly the age that Year 7 students are, they were very risk averse about that concern, that we were going to raise problems.”	Uptake of the intervention – potential barrier
Access to technology	Teacher 602: “The program was probably harder for us to administer this semester due to the 'public works' building going on at school. This meant we were constantly shifting venues, kids didn't always have access to the internet and they stopped bringing devices to class because of this. No devices made it stressful because then you were constantly reverting to 'plan B's' all the time and this creates stress.”	Uptake of the intervention – potential barrier
	Policy personnel 06: “There’s nothing worse for a teacher than thinking they’re doing this once a week for eight weeks, and then losing a lesson because oh, the technology’s working, it’s not working. That’s a huge barrier, especially in remote areas.”	
Heterogeneity of technology	Researcher 02: “So, problem is different schools have different IT setups, and so if we design an app, it may not all work smoothly on every application, especially when you talk about deployment within a scale, enlarged number of schools. I think that would require pretty much a review of what we have built this for. What we have built, this right now, probably it only would be more like a prototype. So there would be definitely much heavier investment in terms of IT to make it work in that scale.”	Uptake of the intervention – potential barrier
Inclusivity	Researcher 04: “The cultural inclusivity was a bit of an issue, as well as just the level of literacy. So, it’s a bit of a passion of mine of making it accessible to a range of intellectual levels or so, with kids with say hearing issues, sight issues and just intellectual disabilities.”	Uptake of the intervention – potential barrier
**Theme: Teacher champions**		
Teacher champions	Teacher 1103: “We were fortunate that I could take over the overseeing of this program and therefor make it easier for the class teachers. I felt that the program was not taken as seriously by class teachers as it could have been BUT they are very busy in their own day to day teaching and other responsibilities so honestly they didn't have time. And it’s the old adage 'if you don't have ownership the delivery/teaching is sometime impeded'.”	Uptake of the intervention – potential enabler
**Theme: Sustainability**		
Staff resources	Researcher 01: “To be sustainable in the long term, it would need people who are—it would need a couple of staff dedicated to it, but a couple of staff could manage it across every school in the country.”	Cost – potential increase in cost in provision of human resources
Funding	Researcher 02: “And we have some other mass programs, has been very successful when they were funded, during the funding period. Of course, when the funds dried up, essentially, they no longer can maintain the program, and either they just go to a commercial company, or they just become zombie programs.”	Cost – sustainability of funding required
**Theme: Potential changes to the intervention for delivery at scale**		
Engagement	Teacher 704: “Students felt that as the platform for the program was online, more of the activities could have been interactive or conducive to a computer rather than slabs of writing they were required to read, and often skip ahead. Their suggestion was even a video of someone reading this information as a starting point for improvement, which a preventative skip ahead button, forcing them to listen and learn all the information.”	Cost – potential increase in cost to further facilitate student engagement with the program
	Researcher 01: “Okay, so we’ve learnt a lot in doing the intervention and I can see that we—and it’s what I would like to do to it, is add an audio track behind the whole program.”	
	Researcher 02: “Just like any software that involves lots of users, there will be different human factors there as well. Like, we didn’t expect students to skip through the content without reading them, so we try to establish how to make the program more effective and use technology in a way to actually guide students to read more carefully, to obtain the knowledge better.”	
Enhanced integration of teacher tracking and assessment tools	Researcher 04: “The feedback also that we received to what teachers from the teaching perspective was, that they didn’t know what their students were answering, they didn’t know how much their students were engaging with the program and considering the program asks the students to consider their daily lives and consider their personal goals and things like that, and ask them to create change around some of those issues, some of the content that the students are answering can be confidential which is why we couldn’t give teachers access to what the students were answering, but we do realise that the teachers—because it is covering some of, I think it was eighty hours of the curriculum, the teachers do need to report and assess on that, so that was something that we had overlooked or perhaps hadn’t addressed as well as we would have liked to initially.”	Cost – potential increase in cost to further integrate teacher tracking and assessment tools into the program
	Researcher 05: “So throughout the main program, I would put in more activities that the teachers can actually get responses to. So some of it might be that the students can still do it as they do it now, so that’s helping them reflect on their behaviours and stuff like that, which teachers won’t have access to. So it encourages students to still do it honestly. But then yeah, have other aspects where teachers can actually use it for marking.”	
Additional student and teacher resources	Researcher 01: “We’ve got a teacher manual that they get. Again, I would say it might be more valuable to develop a video for teachers rather than send them a manual but, give them the manual as well because they can check, go back and look at things. But give them an instruction video would be probably something that, if you were trying to go to scale, I’d definitely do.”	Cost- potential increase in cost to develop such resources

A key perceived benefit of the SHINE intervention when considering scale-up was the opportunity for universal, consistent, evidence-based intervention content through online delivery. The potential for economies of scale emerged as a theme, as the online intervention delivery model provides a relatively low cost mechanism for reaching participants across broad geographical regions. Within the universal delivery model, a key benefit of the intervention was also recognised as the ability to target content to specific students in a confidential way.

However, several contextual themes that might impact on the uptake of the intervention at scale emerged, including potential acceptability and accessibility issues. In terms of acceptability, the busy nature of the school environment and the critical need to integrate the intervention into the existing work practices and curriculum were recurrent themes and key facilitators to potential scale-up.

Sensitivities around intervention content suggest that the intervention in its current form is unlikely to be delivered at scale in all school sectors (i.e. government, catholic and independent secondary schools) (Table 4). The technological environment of Australian secondary schools may also have an impact on intervention reach, and this should also be considered for implementation and scale. Potential barriers to scale-up include access to IT equipment at the student level (including inconsistent access to devices if students leave their individual devices at home when they are required at school), access to IT equipment at the school level (in schools that do not require students to have their own individual devices and instead have limited school IT equipment) and access to a reliable internet connection. In addition, interview participants also discussed the cultural, intellectual and disability inclusivity of the intervention (Table 4).

A number of key alterations to the intervention program emerged as important in supporting and sustaining intervention scale-up. The need to incorporate more engaging content for students to prevent them “clicking” through the program, the addition of more audio and visual components and enhanced integration of teacher tracking and assessment tools within the online delivery platform were recurrent themes that may impact on the costs of intervention development and implementation and associated budgets. One study participant noted that collaboration with an online learning designer to review and revise the program prior to implementation at scale may help to strengthen student and teacher engagement. The engagement of teacher “champions” also emerged as a key theme that was critical for implementation. Within the confines of the cRCT these SHINE champions were identified as liaisons between school staff and the research team. Assuming wider implementation and scale-up, the role for these SHINE champions would become even more important in the feasibility and sustainability of the intervention. There may be associated implementation costs with identifying and training these champions in their roles that were not accounted for within the costing confines of the cRCT.

The need for additional student and teacher resources at scale was also a recurrent sub-theme, with associated impacts on intervention cost at scale. Such resources included additional content for students such as extension or workbook activities that would further consolidate their learning and additional teacher training and professional development opportunities, potentially utilising delivery modes conducive to scale such as instructional videos or online delivery. This may be particularly important in light of one participant’s view that HPE teachers are not always specialised within the HPE field, particularly in older teacher cohorts.

Revision of the IT platforms and infrastructure underpinning the SHINE program emerged as important for it to be reliably delivered at scale, and this would likely have a material implementation cost associated with the human resources required to undertake this review. Interview participants noted that human resources associated with both technical administration and program administration of the intervention would be crucial in ensuring feasibility and sustainability at scale. Within the cRCT administrative time costs were relatively minimal (0.3 FTE year 1, 0.1 FTE in subsequent years) as the number of intervention schools was far less than what would be expected at scale. Within the cRCT most of the Project Manager’s time was spent in co-ordinating the research-related components of the intervention, but at scale more time would be required for intervention delivery support to a much greater number of schools and students. In addition, time costs associated with the development and updating of intervention content would need to be considered to ensure that the intervention continues to reflect best available evidence over time.

Finally, funding was recognised as an important facilitator of intervention scale-up and sustainability by interview participants. Potential funding avenues that emerged from the data included solely government provision, or government partnership with philanthropic organisation/s for implementation and delivery at scale. The dual role of both State and Federal levels of government in education in Australia emerged as a source of complexity if the intervention was implemented and delivered at scale.

## Discussion

The SHINE intervention as conducted within the cRCT represents a relatively low-cost intervention, using a clear delivery pathway (i.e. through education systems by aligning with curriculum requirements) and an economical mode of delivery (i.e. online). Certainly when compared to the limited economic evidence of cost or cost-effectiveness of interventions conducted in a secondary school setting and aiming to improve physical activity and/or healthy eating or reduce obesity, the SHINE intervention is comparatively low-cost [37, 38]. For example, the cost of a 24-month multi-component intervention implemented in secondary schools in New South Wales, Australia was estimated at AUD$394 per participant [38]. Comparing cost or cost-effectiveness between interventions is however challenging, given the substantial heterogeneity in intervention scope, design, target populations, delivery mode, intensity and duration.

To the best of our knowledge, this paper presents the first published cost estimate of a universal education program aiming to improve nutrition, physical activity and wellbeing among secondary school students delivered online. While the effect of the intervention has not yet been established, the proposed economic evaluation [21] will add to the relatively limited evidence base for the cost-effectiveness of web-based obesity prevention interventions [39]. Findings from the qualitative component of this study will enhance the relevance and applicability of this economic evidence [21], providing important information to trialists and policy-makers on potential economic-related impacts of implementation and scale at a relatively early research stage. The findings from this study will be useful, alongside the results from the within-trial and modelled economic evaluation, to inform decisions on whether to progress to a Type III implementation trial. While there are a number of factors that may influence the decision to scale-up an intervention, including political, social, user organisation and resource team attributes [40], the findings presented here will be insightful in considering the costs of scaling up. This knowledge may also help to reduce potential economic-related barriers and enhance economic-related facilitators should the intervention progress from a research setting into policy and practice [41]. This may be particularly important given the increasing focus on research translation and moving evidence-based knowledge into policy and practice [42].

Recent evidence suggests that there is significant scope for further integration of the economic constructs related to implementation and scale in commonly used implementation and scale-up models, theories and frameworks [31]. There is a growing focus on designing public health interventions that are suitable for delivery in real-world conditions [43], and therefore careful consideration should be given to relevant economic constructs early in the research process. To date, quantitative measures alone have been insufficient to strongly predict dissemination, implementation and maintenance outcomes [44]. Given the increasing focus on research translation, we posit that health economists should consider more integration of qualitative data to inform quantitative cost and cost-effectiveness results, specifically focusing on economic constructs related to implementation and scale-up, to generate this real-world, policy-relevant evidence. Our findings also demonstrate the clear need for health economists to work more closely with trialists and implementation scientists from early in the research process, so that rigorous economic evidence on important factors such as costs, resources and funding can be collected to inform both implementation and scale as research progresses [45].

Qualitative findings from our study suggest that there may be substantial variation in intervention cost between that experienced within the controlled research setting and what might be experienced if the SHINE intervention was scaled-up and delivered more widely. This is not unexpected, given that levels of research funding and the degree to which they reflect the actual full economic cost of developing and testing interventions varies [46]. Importantly, curriculum experts were consulted in the development of SHINE intervention content and so there is likely to be minimal additional cost of curriculum alignment should the intervention be scaled-up for delivery. Intervention costs associated with program administration within the cRCT were relatively minimal, and are likely to be higher should the intervention be delivered at scale. Intervention costs associated with technical administration and delivery of the program from an IT expert were not included in our within-trial cost analysis, as these costs for the purpose of the cRCT were related to intervention development. Once the IT components were developed they did not require an IT expert to maintain them for the purposes of the relatively short intervention period within the cRCT. At scale, technical expertise to maintain and update the intervention would be required, and these costs should be considered within future analyses. Qualitative findings also suggest the need for additional student and teacher resources at scale which would impact on intervention cost, although presumably to a lesser degree.

Qualitative findings suggest significant implementation costs would likely be experienced in order for SHINE to be scaled up and delivered to populations effectively and sustainably. Revision of intervention content so that it is more accessible to students of diverse backgrounds and with varying educational and ability levels may be an important consideration of delivery at scale. Adaptation of intervention content (for example, targeting children with low literacy levels) may incur additional development costs which, while not impacting on intervention cost-effectiveness when estimated as steady state (i.e. fully operational and excluding development costs), may impact on implementation costs and associated budgets. While these implementation costs do not impact cost-effectiveness at scale, they could have material budgetary, and affordability impacts and so require careful planning and consideration. This is particularly important given that decision-makers often report that implementation costs are an important factor in lack of widespread adoption of public health interventions [47, 48]. The systematic review by Reeves et al. [49] identified only 14 economic evaluations of implementation strategies of public health interventions. Of the 14 included studies, only two were interventions to improve obesity-related behaviours (i.e. physical activity alone [38] and physical activity and health eating behaviours [50]. There is extremely limited published evidence on the cost of implementation strategies for scale-up of obesity prevention interventions within school or educational settings [51]. Clearly there is a need for more research in this area and, should the SHINE intervention progress to a Type III translational study, it will be important to track and document these costs of implementation in order to build this evidence.

In addition, the revision of intervention content may result in increased uncertainty of intervention effect if changes are material or if the intervention population differs markedly from that within the cRCT. While intervention schools were located in local government areas spanning a range of Socio-Economic Index for Areas Index of Relative Socio-economic Disadvantage scores (SEIFA IRSD; Table 1), the majority of intervention schools were located in a metropolitan area with a SEIFA IRSD of 7 or above (indicating relatively less disadvantage than areas with lower scores). This may impact estimates of cost-effectiveness at scale and should be considered within analyses. In addition, it is possible that the reach of the intervention at scale may only extend as far as government and potentially some independent secondary schools, and estimates of cost and cost-effectiveness at scale should factor this into analyses. Finally, education sector funding complexity suggests time and resources would be required to co-ordinate between potential program providers for delivery at scale, impacting on both implementation costs as funding and delivery pathways are developed, and potentially intervention costs related to stakeholder management over the longer term.

Strengths of this study include the approach taken, using both qualitative and quantitative methods, and the comprehensive, prospective tracking of SHINE intervention costs alongside the Type II cRCT. Limitations of this study include the inability to interview teacher stakeholders to the SHINE intervention due to government pandemic restrictions. We attempted to circumvent this limitation through the inclusion of teacher survey responses but recognise this does not allow for the expected richness of semi-structured interview data. We suggest further studies incorporate the unique stakeholder perspective of the teachers to the SHINE intervention, so that future estimates of scale can more fully incorporate this perspective.

## Conclusions

Within the confines of the Type II translational research trial, SHINE is a low-cost intervention. Findings from our study however suggest that the cost of the intervention if delivered at scale will be higher, in part due to increased IT and administrative time costs and exacerbated by a potentially limited reach of the intervention across all school sectors. Findings also suggest significant implementation costs associated with program adaptation for delivery at scale, and the resources and funds required to undertake this adaptation should be considered within resource allocation decisions. More broadly, our study findings demonstrate the utility of combining quantitative and qualitative economic data to better understand the implications of implementation and scale-up on intervention budgets and cost-effectiveness as research moves to practice.

## Supplementary Information

**Additional file 1.** Semi-structured interview guides.

**Additional file 2.** CORE-Q checklist.

**Additional file 3.** Results from sensitivity analysis.

## Data Availability

The datasets used and/or analysed during the current study are available from the corresponding author on reasonable request.
