# Supplementary material for: Exploring the economics of public health intervention scale-up: a case study of the Supporting Healthy Image, Nutrition and Exercise (SHINE) cluster randomised controlled trial
Source: BMC Public Health. 2022 Jul 14;22:1338. doi: 10.1186/s12889-022-13754-0 (PMC9281014; doi:10.1186/s12889-022-13754-0)
Supplement: Supplementary file 1 — Additional file 1. Semi-structured interview guides. [file 12889_2022_13754_MOESM1_ESM.pdf]

Exploring the economics of public health intervention scale-up: a case study of the Supporting Healthy Image, Nutrition and Exercise (SHINE) cluster randomised controlled trial.

Vicki Brown[1], Huong Tran[1], Joanne Williams[2], Rachel Laws[3], Marj Moodie[1]

1 Deakin University, Geelong, Deakin Health Economics, Institute for Health Transformation, Global Obesity Centre (GLOBE), School of Health and Social Development, Victoria 3220, Australia
2 Swinburne University of Technology, School of Health Sciences, Hawthorn, Victoria 3122, Australia
3 Deakin University, Geelong, Institute for Physical Activity and Nutrition, Victoria 3220, Australia

Corresponding author: Dr Vicki Brown, Deakin University, Geelong, Deakin Health Economics, Institute for Health Transformation, Global Obesity Centre (GLOBE), School of Health and Social Development, Victoria 3220, Australia. victoria.brown@deakin.edu.au

Additional File 1- Semi-structured interview guides

CIs/AIs and project staff

Introduction:

Thanks for taking the time to talk with me today.

Did you have a chance to read the information sheet?

You were invited today for interview because of your involvement as a [CI/AI/Researcher/Project Manager] with the SHINE intervention.

The purpose of the interview today is to hear your thoughts on the SHINE intervention, and how it might look and work if the intervention was "scaled-up" and incorporated into schools more broadly.

Are you happy for us to tape record the interview to ensure we have an accurate record of your comments and for the purposes of analysis? We will not identify any individuals or institutions in reporting of the interview findings.

First, I would like to hear about you and your thoughts on the SHINE intervention as it is being delivered in the RCT.

1. To start with, can you tell me a little bit about your background and your role in the SHINE RCT?

2. What would you say are the key strengths of the SHINE intervention?

3. What would you say are the key weaknesses of the SHINE intervention?

Prompts:

- Complexity
- Design
- Strength of evidence
- Appeal to teachers, students, potential providers

4. What, if any, were the potential facilitators to the initial engagement of schools in the SHINE intervention? By initial engagement, I mean the phase of time where schools were being recruited and getting ready to deliver the intervention. What, if, any, were the potential facilitators in preparing those school settings to deliver the intervention?

   What, if any, were the potential barriers to the initial engagement of schools in the SHINE intervention?

5. What, if any, were the potential facilitators to the implementation of the SHINE intervention within the school setting? By implementation, I mean the phase of time where the intervention was being delivered.

   What, if any, were the potential barriers to the implementation of the SHINE intervention within the school setting?

6. What, if any, were the potential facilitators to the sustainability of the SHINE intervention within the school setting (during the trial)?

   What, if any, were the potential barriers to the sustainability of the SHINE intervention within the school setting (during the trial)?

7. Do you think SHINE is a low cost, medium cost or high cost intervention? Why?

   Prompts:

   - Perceived cost drivers?

I want you to think about the SHINE intervention now, and whether it might be suitable for wider delivery at scale (i.e. more broadly across all high schools, in the "real world" and outside of a research setting). My next questions are on the scalability of the intervention.

8. How scaleable do you think the SHINE intervention is? Do you think it is suitable for more wide spread implementation to all schools? Why/ why not?

   Prompts:

   - Envisioned reach (public/private-non-public, state/national)

9. What aspects of the SHINE intervention, if any, makes it more or less amenable to delivery at scale?

10. What would be required for delivery of the intervention at scale and in the "real world"? How do you imagine the SHINE intervention could be scaled up? What would be your key recommendations for successful scale up of SHINE?

Prompts:

- Approach to scale up (e.g. curriculum (policy), voluntary adoption etc)
- Resourcing
- Support
- Outgrows research, to be embedded into the system
- Vertical approach (simultaneous; involves the introduction of an intervention simultaneously across a whole system and results in institutionalisation of a change through policy, regulation, financing or systems change);
- Horizontal approach (stepwise; involves the introduction of an intervention across different sites or groups in a phased manner, often beginning with a pilot program, followed by stepwise expansion with refinements along the way)
- Ownership of the intervention
- Funding
- Fidelity at scale
- Design
- Alignment with curriculum

- Alignment with school processes

11. What changes to the initial engagement of schools would be required for delivery at scale, if any?

12. What changes to the actual intervention would be required for delivery at scale, if any?

Prompts:

- Intervention content and core components

- Method for intervention delivery

- Intervention support/maintenance/administration

13. What extra resources, if any, would be required for delivery of SHINE at scale (as compared to within a research environment)?

    Prompts:
- Equipment, funding, time
- Do you think these resources will be available? If not, which resources won't be available?

14. As SHINE is currently in the RCT phase, we don't yet know the intervention effect. Can you hypothesise whether SHINE might be more effective, less effective or of about the same effect as within the RCT if delivered at scale? Can you elaborate on why?

15. Can you hypothesise whether SHINE might be more costly, less costly or of about the same cost as within the RCT if delivered at scale? Can you elaborate on why?

16. Can you think of any precedents, where an intervention like SHINE has been successfully scaled up within the school setting?

    If so, can you reflect on what about that intervention made it appropriate for scale-up and more widespread adoption?

That is all the questions I have. Would you like to add anything else?

Thank you for your time. Your contribution today can be used to improve the intervention and will help to better understand the potential for scale-up of SHINE.

Potential program providers

Introduction:

Thanks for taking the time to talk with me today.

Did you have a chance to read the information sheet?

The purpose of the interview today is to hear your thoughts on the potential scalability of the Supporting Health Image, Nutrition and Exercise (SHINE) intervention.

Are you familiar with the SHINE intervention? (information sheet)

SHINE is an intervention that is currently being tested in an RCT in Victorian high schools. SHINE replaces the usual Health and Physical Education (HPE) curriculum for eight weeks and is a self-directed, online learning program. SHINE uses an individually tailored body image and weight management program for Year 7 students, and delivers universal education about healthy nutrition and physical activity to adolescents.

Are you happy for us to tape record the interview to ensure we have an accurate record of your comments and for the purposes of analysis?

We will not identify any individuals in reporting of the interview findings.

1. SHINE is currently being trialled in a number of Victorian high schools. From your perspective, what would be the essential ingredients for an intervention like SHINE to be successfully delivered in the high school setting?

   And perhaps can't answer if no prior knowledge of SHINE – do you think SHINE has these factors?

2. How feasible do you think the wide-scale delivery of an intervention like SHINE is within the current school environment?

Prompts:

If feasible – why. If not feasible – why not.

- Resourcing
- Budgeting
- Support/administration/maintenance
- Delivery workforce
- Alignment with curriculum
- What about differences between states and territories?
- What about between school systems: catholic vs public vs independent

3. How acceptable do you think the wide-scale delivery of an intervention like SHINE would be?
   To schools (principals, teachers)
   To parents
   To stakeholders (e.g. department, curriculum authority etc).

4. In your opinion, are there factors that might facilitate the implementation of an intervention like SHINE within the school setting?

5. In your opinion, what would be the barriers to implementation of an intervention like SHINE within the school setting?

Prompts for 3 and 4:

- Similar programs/measures already in place that might facilitate or hinder scale up
- Funding
- Resourcing
- Support
- Complexity
- Quality
- Content and "fit" with setting, context
- Delivery method and "fit" with setting, context

6. What might be the potential benefits of wide-scale delivery of an intervention like SHINE, if any?

7. What might be the potential limitations or drawbacks of wide-scale delivery of an intervention like SHINE, if any?

8. How would you envision delivery at scale of an intervention like SHINE?

Prompts:

- Ownership
- Funding
- Envisioned reach (public/non-public, state/national)
- Vertical approach (simultaneous; involves the introduction of an intervention simultaneously across a whole system and results in institutionalisation of a change through policy, regulation, financing or systems change);
- Horizontal approach (stepwise; involves the introduction of an intervention across different sites or groups in a phased manner, often beginning with a pilot program, followed by stepwise expansion with refinements along the way)
- Who would likely be involved in such a process? What steps would likely be required?

9. Can you think of any precedents, where an intervention like SHINE has been successfully scaled up within the school setting? If so, can you reflect on what about that intervention made it appropriate for scale-up and more widespread adoption?

That is all the questions I have. Would you like to add anything else?

Thank you for your time. Your contribution today can be used to improve the intervention and will help to better understand the potential for scale-up of SHINE.

Teachers

Introduction:

Thanks for taking the time to talk with me today.

Did you have a chance to read the information sheet?

You were invited for interview today because of your experience with the SHINE intervention.

The purpose of the interview today is to hear your thoughts on the SHINE intervention, and how it might be “scaled-up” and incorporated into schools more broadly (i.e. delivered across all high schools).

Are you happy for us to tape record the interview to ensure we have an accurate record of your comments and for the purposes of analysis? We will not identify any individuals or schools in reporting of the interview findings.

First, I would like to hear about your experiences in implementing the SHINE intervention in your school

1. To start with, can you tell me a little bit about your background and your role in implementing SHINE in your school?

Prompts:

- How did you become involved with SHINE?

2. What would you say are the key strengths of the SHINE intervention?

3. What would you say are the key weaknesses of the SHINE intervention?

4. Were there any things that made it easier or more difficult to implement SHINE in your school?

Prompts:

- Tell me more about that
- How did you manage these challenges
- Resources
- Time / competing demands
- Support at school level
- Student interest
- Methods for engagement
- Any modifications they may have made

5. How easy or difficult did you find it to incorporate SHINE into your existing workplans and teaching processes? Can you tell me a bit about how you did that?

Prompts:

- Curriculum
- Planning time, teaching time
- Small or large departure from current practices?

6. How effective (if at all) do you think SHINE has been at your school?

Prompts:

- At the student level: primary outcomes- weight, weight and shape concerns; secondary outcomes – increased PA, decreased sedentary behaviour, improved dietary intake, increased quality of life. Engagement, satisfaction.
- At the individual classroom teacher level – e.g. satisfaction with program
- At the school level

7. Are there any changes that you would suggest to the SHINE intervention?

Prompts:

- Changes to the intervention itself?
- Changes to the way the intervention is implemented/supported?

8. How well equipped did you feel with the skills and resources to deliver SHINE?

Prompts:

- At the individual level
- School level support
- What do you think you would need to feel more equipped – skills, resources, other……

I want you to think about the SHINE intervention now, and whether it might be suitable for wider delivery at scale (i.e. more broadly across all high schools).

9. What aspects of the SHINE intervention, if any, makes it amenable to delivery at scale?

10. What about the SHINE intervention, if anything, makes it challenging to be delivered at scale? How would you suggest those challenges be overcome?

Prompts for 10 and 11:

- Ownership of the intervention
- Alignment with curriculum

- Alignment with school processes
- What extra resources, if any, might be required?
- How suitable, if at all, would it be to deliver SHINE in a diverse range of schools (such as schools in rural areas, schools in low SEP areas)? Or to a diverse body of students?

11. Do you think that scale up of SHINE would result in the intervention being more effective, less effective or of about the same effect as within the RCT of which you have been a part? Can you elaborate on why?

12. Do you think that SHINE might be more costly, less costly or of about the same cost as within the RCT if delivered at scale? Can you elaborate on why?

13. What would be your key recommendations for successful scale up of SHINE?

Prompts:

- Approach to scale up (e.g. curriculum (policy), voluntary adoption etc)
- Resourcing
- Support

14. Can you think of any precedents, where an intervention like SHINE has been successfully scaled up within the school setting?
    If so, can you reflect on what about that intervention made it appropriate for scale-up and more widespread adoption?

That is all the questions I have. Would you like to add anything else?

Thank you for your time. Your contribution today can be used to improve the intervention and will help to better understand the potential for scale-up of SHINE.
