# Supplementary material for: Exploring the economics of public health intervention scale-up: a case study of the Supporting Healthy Image, Nutrition and Exercise (SHINE) cluster randomised controlled trial
Source: BMC Public Health. 2022 Jul 14;22:1338. doi: 10.1186/s12889-022-13754-0 (PMC9281014; doi:10.1186/s12889-022-13754-0)
Supplement: Supplementary file 2 — Additional file 2. CORE-Q checklist. [file 12889_2022_13754_MOESM2_ESM.pdf]

Exploring the economics of public health intervention scale-up: a case study of the Supporting Healthy Image, Nutrition and Exercise (SHINE) cluster randomised controlled trial.

Vicki Brown[1], Huong Tran[1], Joanne Williams[2], Rachel Laws[3], Marj Moodie[1]

1 Deakin University, Geelong, Deakin Health Economics, Institute for Health Transformation, Global Obesity Centre (GLOBE), School of Health and Social Development, Victoria 3220, Australia

2 Swinburne University of Technology, School of Health Sciences, Hawthorn, Victoria 3122, Australia

3 Deakin University, Geelong, Institute for Physical Activity and Nutrition, Victoria 3220, Australia

Corresponding author: Dr Vicki Brown, Deakin University, Geelong, Deakin Health Economics, Institute for Health Transformation, Global Obesity Centre (GLOBE), School of Health and Social Development, Victoria 3220, Australia. victoria.brown@deakin.edu.au

Additional File 2- CORE-Q checklist [1]

CORE-Q checklist [1]

No.	Item	Guide questions/description	Page no. in manuscript
Domain 1: Research team and reflexivity			
1	Interviewer/facilitator	Which author/s conducted the interview or focus group?	Page 9
2	Credentials	What were the researcher's credentials? E.g. PhD, MD	Page 9
3	Occupation	What was their occupation at the time of the study?	Page 9
4	Gender	Was the researcher male or female?	Page 9
5	Experience and training	What experience or training did the researcher have?	Page 9
Relationship with participants			
6	Relationship established	Was a relationship established prior to study commencement?	Page 9
7	Participant knowledge of the interviewer	What did the participants know about the researcher? e.g. personal goals, reasons for doing the research	Page 9
8	Interviewer characteristics	What characteristics were reported about the interviewer/facilitator? e.g. Bias, assumptions, reasons and interests in the research topic	Page 9-10
Domain 2: study design			
Theoretical framework			
9	Methodological orientation and Theory	What methodological orientation was stated to underpin the study? e.g. grounded theory, discourse analysis, ethnography, phenomenology, content analysis	Page 8-10
Participant selection			
10	Sampling	How were participants selected? e.g. purposive, convenience, consecutive, snowball	Page 10-11
11	Method of approach	How were participants approached? e.g. face-to-	Page 9

		face, telephone, mail, email	
12	Sample size	How many participants were in the study?	Page 9-10, 13
13	Non-participation	How many people refused to participate or dropped out? Reasons?	Page 13
Setting			
14	Setting of data collection	Where was the data collected? e.g. home, clinic, workplace	Page 9-10, 13
15	Presence of non-participants	Was anyone else present besides the participants and researchers?	Page 9-10
16	Description of sample	What are the important characteristics of the sample? e.g. demographic data, date	Page 13, Table 2
Data collection			
17	Interview guide	Were questions, prompts, guides provided by the authors? Was it pilot tested?	Page 9-10, Additional File 1
18	Repeat interviews	Were repeat interviews carried out? If yes, how many?	NA
19	Audio/visual recording	Did the research use audio or visual recording to collect the data?	Page 9-10
20	Field notes	Were field notes made during and/or after the interview or focus group?	NA
21	Duration	What was the duration of the interviews or focus group?	Page 13
22	Data saturation	Was data saturation discussed?	No
23	Transcripts returned	Were transcripts returned to participants for comment and/or correction?	No
Domain 3: analysis and findings			
Data analysis			
24	Number of data coders	How many data coders coded the data?	Page 9-10
25	Description of the coding tree	Did authors provide a description of the coding tree?	No

26	Derivation of themes	Were themes identified in advance or derived from the data?	Page 9-10
27	Software	What software, if applicable, was used to manage the data?	Page 9-10
28	Participant checking	Did participants provide feedback on the findings?	No
Reporting			
29	Quotations presented	Were participant quotations presented to illustrate the themes / findings? Was each quotation identified? e.g. participant number	Page 14-18
30	Data and findings consistent	Was there consistency between the data presented and the findings?	Yes
31	Clarity of major themes	Were major themes clearly presented in the findings?	Yes
32	Clarity of minor themes	Is there a description of diverse cases or discussion of minor themes?	Yes

REFERENCES

1. Tong A, Sainsbury P, Craig J. Consolidated criteria for reporting qualitative research (COREQ): a 32-item checklist for interviews and focus groups. International journal for quality in health care. 2007;19(6):349-357.
