# Supplementary material for: Exploring the economics of public health intervention scale-up: a case study of the Supporting Healthy Image, Nutrition and Exercise (SHINE) cluster randomised controlled trial
Source: BMC Public Health. 2022 Jul 14;22:1338. doi: 10.1186/s12889-022-13754-0 (PMC9281014; doi:10.1186/s12889-022-13754-0)
Supplement: Supplementary file 3 — Additional file 3. Results from sensitivity analysis. [file 12889_2022_13754_MOESM3_ESM.pdf]

Exploring the economics of public health intervention scale-up: a case study of the Supporting Healthy Image, Nutrition and Exercise (SHINE) cluster randomised controlled trial.

Vicki Brown[1], Huong Tran[1], Joanne Williams[2], Rachel Laws[3], Marj Moodie[1]

1 Deakin University, Geelong, Deakin Health Economics, Institute for Health Transformation, Global Obesity Centre (GLOBE), School of Health and Social Development, Victoria 3220, Australia

2 Swinburne University of Technology, School of Health Sciences, Hawthorn, Victoria 3122, Australia

3 Deakin University, Geelong, Institute for Physical Activity and Nutrition, Victoria 3220, Australia

Corresponding author: Dr Vicki Brown, Deakin University, Geelong, Deakin Health Economics, Institute for Health Transformation, Global Obesity Centre (GLOBE), School of Health and Social Development, Victoria 3220, Australia. victoria.brown@deakin.edu.au

Additional File 3- Results from sensitivity analysis

Results from sensitivity analysis

Assuming that teacher training and assistance was provided in a one hour in-person session with an intervention researcher to all schools, with all intervention group teachers participating; excluding teacher time costs for lesson planning.

**Time cost**	
Year 1	
Project Management, intervention delivery co-ordination	$30 335
Teacher training and assistance	$6 286
Year 2	
Project Management, intervention delivery co-ordination	$8 049
Year 3	
Project Management, intervention delivery co-ordination	$8 049
TOTAL TIME COST	$52 719
TOTAL TIME COST (discounted)	$52 022
**Travel costs**	
Year 1	
Travel to provide teacher training and assistance	$1 515
TOTAL TRAVEL COST	$1 515
**Equipment costs**	
Year 1	
Teacher training manuals	$428
Custom earbuds	$3 585
TOTAL EQUIPMENT COST	$4 013
TOTAL COST	$58 246
**TOTAL COST (discounted)**	**$57 550**
**TOTAL INTERVENTION COST PER STUDENT (discounted)**	**$26**

Table notes: Costs are in 2021 Australian dollars. Negative values indicate cost-savings.
